# Wild food plants and minor crops in the Ripollès district (Catalonia, Iberian Peninsula): potentialities for developing a local production, consumption and exchange program

**DOI:** 10.1186/s13002-016-0122-y

**Published:** 2016-10-20

**Authors:** Montse Rigat, Airy Gras, Ugo D’Ambrosio, Teresa Garnatje, Montse Parada, Joan Vallès

**Affiliations:** 1 Laboratori de Botànica - Unitat Associada CSIC, Facultat de Farmàcia i Ciències de l’Alimentació, Universitat de Barcelona, Av. Joan XXIII s.n, 08028 Barcelona, Catalonia Spain; 2 Institut Botànic de Barcelona (IBB-CSIC-ICUB), Passeig del Migdia s.n., Parc de Montjuïc, 08038 Barcelona, Catalonia Spain

**Keywords:** Demographic factors, Ethnobotany, Minor crops, Pyrenees, Wild edible plants

## Abstract

**Background:**

Wild food plants (WFP) have always been consumed by humans, first as the main basis of their food and, since the origins of agriculture, as ingredients of normal diets or as an alternative during situations of scarcity. In contemporary industrialized societies their use is for the most part being abandoned, but they may still play an important role. With the purpose of advancing in the ethnobotanical knowledge of one region of the Catalan Pyrenees, the present study reports the findings of a research project conducted in the Ripollès district (Catalonia, Iberian Peninsula), concerning ethnobotanical knowledge and use of wild and semi-wild vascular plants as foods, along with minor crops.

**Methods:**

From August 2004 to July 2014, we performed 104 interviews (93 of which yielded data on food plants) with 163 informants, using the method of semi-structured ethnobotanical interview. We identified the plants quoted and kept herbarium vouchers.

**Results:**

We detected 967 use reports for 80 wild or naturalized taxa, which are or have been consumed in the Ripollès district, the most cited being *Taraxacum dissectum*, *Cynara cardunculus* and *Origanum vulgare*. Certain frequently reported species such as *Molopospermum peloponnesiacum* and *Taraxacum dissectum* have only been rarely cited previously or indicated as food plant in very restricted geographical areas. Most cited families included Asteraceae and Lamiaceae, followed by Rosaceae and Apiaceae. Preferred consumed plant parts included leaves, followed by aerial parts, along with fruits and infructescences, while most wild food plants are eaten raw or used as condiments. Demographic factors such as age and locality of informants seem to be more relevant to wild food plant knowledge than gender. Middle-aged people and inhabitants from the Higher Freser River Valley seem to have a greater knowledge of WFP, both in relation to the number of species elicited, as well as the diversity of uses and preparations. To a lesser degree, women seem to have a slightly higher WFP knowledge than men. The consumption of these resources is still fairly alive amongst the populace, yet changes affecting younger generations–in most cases abandonment–have been reported by various participants.

**Conclusion:**

The information provided by this kind of research permits the detection of those traditional species that could constitute the basis for the future development and management of wild edible plant resources along with minor crops. It also helps to determine the factors affecting their use, as well as the distinct target groups that such programmes could be addressed to.

## Background

Wild food plants, including semi-wild plants and minor crops (hereafter WFP), have always been consumed by humans, first as the main basis of their food and, since the origins of agriculture, as ingredients of normal diets or as an alternative during situations of scarcity [1, 2]. In various contemporary industrialized societies their use is for the most part being abandoned [3]. Nevertheless, WFP may still play an important role in very different societies in terms of habitual (even necessary, in some cases) consumption and small-scale, familiar trade [4–6]. Moreover, an increased interest has been noticed in Europe, the Mediterranean region and elsewhere on the study and promotion of WFP and non-cultigens production, consumption and exchange (e.g., [7–13]). The study of such species and its associated knowledge is particularly interesting from the ethnobotanical and conservationist points of view [14]. In this sense, knowledge can be defined as “the way people understand the world, and interpret and apply meaning to their experiences” [15].

In some cases, food plants are also used as medicines. This fact, which has reached the industry and is now very popular in the form of healthy products marketed under the name of functional foods or nutraceuticals, also has a clear folk basis [16, 17]. The distinction between food and medicine is often blurred, and many foods are preferably consumed for their healthy properties. This is perhaps more pronounced in less industrialized areas (where the degree of preservation of folk biodiversity uses is, in general, larger), but it is, nonetheless, characteristic everywhere (see, for instance, [6, 12, 18–26]).

Behind the use (or non-use) of WFP, there are many factors, with sociocultural and economic forces playing an important role. Industrialized countries live a new phenomenon associated with WFP and new trends in nutrition and healthy food, in which WFP are transformed from famine foods to delicatessens [27], not only on the average table, but in *haute cuisine* too. One of the founding–and persisting–dishes of French (and the World’s) *nouvelle cuisine*, the salmon escalope with common sorrel, was created in 1963 thanks to a sauce elaborated with a wild herb (*Rumex acetosa*), furnished to Jean and Pierre Troisgros by their mother, who had a folk-based knowledge of its use [28]. Since then, this convergence of innovation and tradition has persisted in haute cuisine with an increasing number of Michelin-starred restaurants using this kind of ingredients [29]. Moreover, the collaboration between representatives of haute cuisine and gastronomy, on the one hand, and of the academia on the other, is currently perceived as necessary. A project in which the authors of the present paper are involved together with the chef Ferran Adrià’s team, aiming to achieve a consensual classification of gastroculinary products [30], constitutes a good example of this cooperation.

Additionally, many chefs and cooks (at different levels, from more popular to more exclusive) have adopted the *terroir*’s cuisine, recently revisited and updated as the local food movement, as well as kilometre 0, slow food or proximity and sustainable cuisine [31]. Just to quote a few examples, the French chef Jean-Paul Jeunet, with two Michelin stars, regularly uses more than 40 wild plant species in his elaborations [32], and the Catalan cook Iolanda Bustos bases her dishes on wild plants, present in absolutely all of them [33]. This is most probably the opportune time to get back to traditional uses of plants, aiming to preserve–and to use–food culture and ethnobotanical knowledge.

Ethnobotany of food plants is, as has been shown, a rather well-developed research field in all kinds of geographical areas and social communities. Mountain areas have always been a particular object of study not only with floristic and landscape focuses, but also with sociological and ethnological ones too [34, 35]. In Europe, abundant ethnobotanical work has been conducted in the Alps and the Balkans (e.g., [19, 23, 36–40]). The Pyrenees have been intensively studied from this point of view too. A pioneer study limited to folk plant names in different Pyrenean languages [41], was later followed by another, focused on medicinal plants, in the Aragonian Pyrenees, but also with folk names in several languages and information on food uses [42]. More recently, research has been carried out in the Basque and Navarran areas [43–47] with medicinal and/or food approaches. Some ethnoecological investigations, basically pointing to food plants, record information from several Iberian mountain zones, including the Pyrenees [48–50]. Finally, the Catalan part of this mountain range is rather well known from an ethnobotanical point of view, with contributions devoted to medicinal, food and other aspects [11, 20, 51–63].

This study complements the article published by our research group on the ethnobotany of food plants with medicinal properties in the Higher River Ter Valley [20] with an extension of the study area and a larger focus. The aims of the present investigation are: i) to provide detailed ethnobotanical information on WFP from the studied area; ii) to report the medicinal use of the reported plants which could then meet the concept of folk functional foods; and iii) to deduce from the results obtained those species that could constitute the basis for local WFP development, and the demographic factors that should be considered in order to successfully undertake such a programme.

## Methods

### Study area

**Fig. 1:**
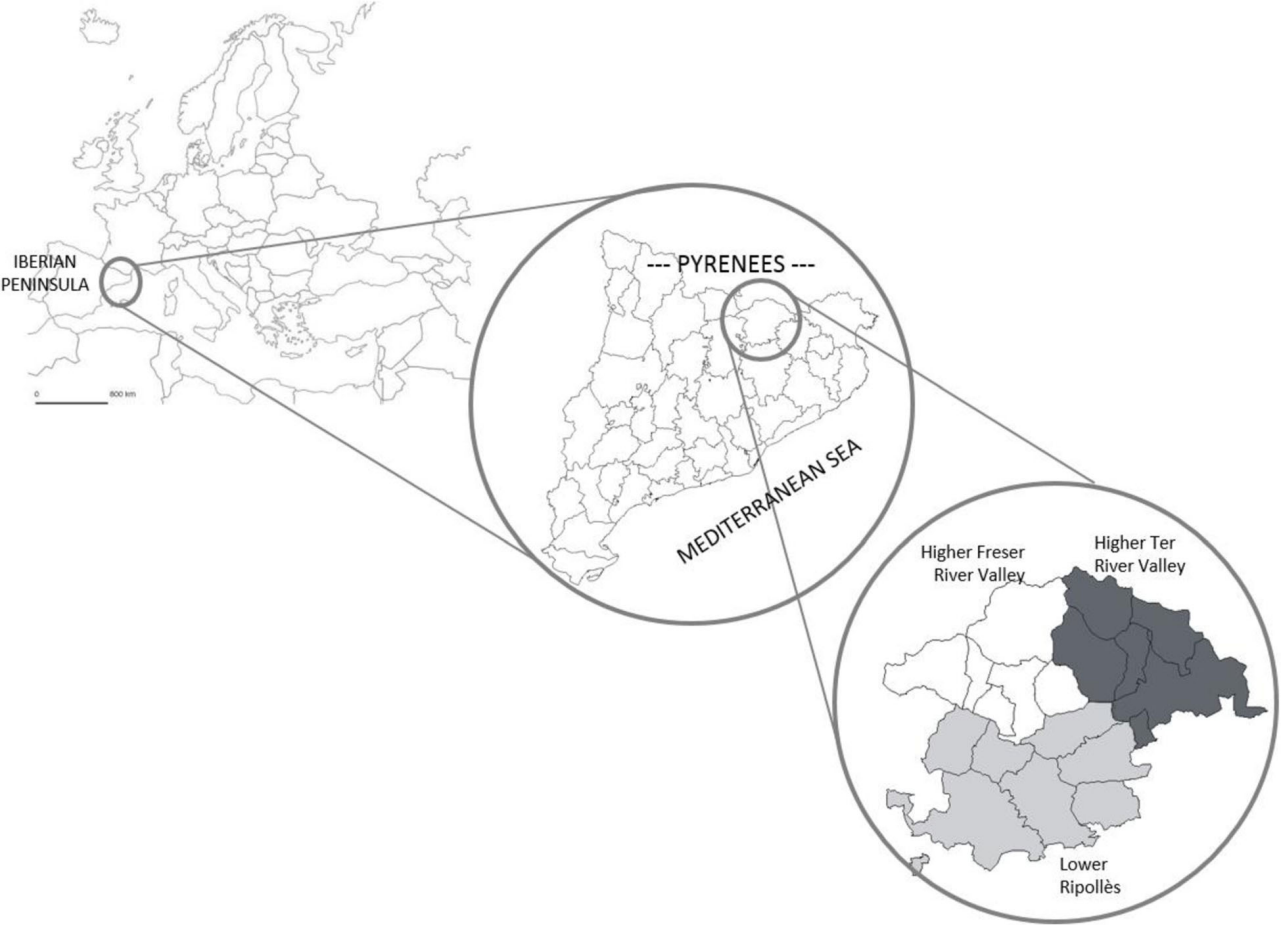
Study area including the three regions conforming the Ripollès district: Higher Ter River Valley, Higher Freser River Valley and Lower Ripollès. Source: Municipis i comarques de Catalunya (http://www.municat.gencat.cat)

The Ripollès district (Fig. 1) is a Catalan territory situated in the eastern Pyrenees (Catalonia, Iberian Peninsula), covering an area of 956.6 km^2^, with a population—in 2014—of 25,700 inhabitants [64] distributed across 19 municipalities, and with a considerable percentage of the residents inhabiting small villages and isolated houses. Despite the fact that agriculture has been replaced by tourism as the main activity of the region, it could be, nevertheless, considered the secondary source of income for most inhabitants, with many farms and houses having their own homegardens for household consumption [62].

Geographically, three distinctive areas constitute the study area: the Higher Ter River Valley, the Higher Freser River Valley and the Lower Ripollès, at the confluence of both rivers (Fig. 1). The north is characterized by a high mountain terrain constituted by the higher Ter and Freser river valleys, and the south, the Lower Ripollès, characterized by middle-range mountains and the confluence of both river valleys into a plain. Cold winters (minimum temperature −1.8 °C and maximum 10.9 °C) and average annual precipitations of 1116.2 mm [64] are characteristic of the high mountainous climate, although in the Ripollès this is softened by its proximity to the Mediterranean Sea. Vegetation is basically alpine and subalpine, defined at higher altitudes by alpine meadows followed by communities with tree species such as *Abies alba* and *Pinus mugo* subsp. *uncinata*, and woodlands with *Fagus sylvatica* and *Quercus* sp. amongst others [65].

As archeological evidence indicates, the Ripollès region has been inhabited, at least, since lower Paleolithic times (ca. 150.000 years ago) [66]. Despite its geomorphologic unity, the region has undergone a disperse history in administrative terms, belonging to the Cerdanya, Osona and Besalú counties depending on the epoch. Its disperse settlement pattern is at least a legacy from medieval times, which has seen relatively little change until recent decades. Textile and metallurgic industries in the lower parts of the district, combined with animal husbandry at higher altitudes, common at the turn of the previous century, have slowly been transformed into rural tourism projects, ski resorts during winter, and other third sector services at the turn of the millennium.

### Methods

To cover aims i and ii, we used semi-structured interviews [67] as a technique for obtaining information from research participants. All interviews were carried out and recorded after prior informed consent was obtained, being executed from August 2004 to July 2014. Most informants were middle aged to elderly people, who were selected on a snowball basis [68] and who were interviewed in the local language, i.e., in the Catalan language, common to interviewers and interviewees. All of them were born in the studied area or have lived there a very significant part of their lives, and most of them have only primary studies and professions linked to agricultural and cattle raising activities. According to the code of ethics of the International Society of Ethnobiology [69], the interviewees’ were asked to give their informed consent to participate in the survey, to register the interviews, to take pictures and to use their information and images.

In some cases group interviews were partaken, yet individualized interviews were the most common. During or after the interview, plant collection was frequently carried out together with the informants, often around their houses or their homegardens, to confirm the identity of the cited species. When collection of the plants accompanied by the informants was not possible, specimens gathered by ourselves in the region were shown to them, also with the purpose of confirming their identity. For this study only vascular plants were considered. Mushrooms, which are highly appreciated in the area (and largely consumed in terms of volume, but involving only a half-dozen taxa) have not been included here, as people did not refer to them when talking about “plants”. Wild, autochthonous and allochthonous plants—some of them naturalised or subspontaneous—have been included, as well as some cultivated plants which do not properly constitute food crops or are marginal and underutilised crops.

All interviews were digitalized into a database and all reported plants were collected and identified using *Flora manual dels Països Catalans* [70]. For botanical families, Angiosperm Phylogeny Group criteria were used ([71] http://www.mobot.org/MOBOT/research/APweb). A voucher for each taxon was prepared and deposited at the herbarium BCN (*Centre de Documentació de Biodiversitat Vegetal*, University of Barcelona). Quantitative analyses included descriptive statistics (mean, standard deviations, ranges) for botanical variables (species, families, part used and mode of preparation) as well as for demographic ones (age, gender, locality). Also the informant consensus factor (F_IC_, the quotient between the number of use reports minus the number of used taxa and the number of use reports minus one; [72]) was calculated to evaluate the consistency and reliability of the information recorded.

To evaluate the tradition of WFP used, we compared the results with food plants contained in two seminal works of Catalan cuisine: a very old one [73] and a very modern one, in two complementary editions [74, 75]. The first one is a compendium of agricultural practices and organisms that was very popular from 17th to 19th centuries, comprising the canon of food plants; more dedicated to cultivated plants, it contained wild ones as well. The second one intends to be the current canon of cuisine, also including more cultivated plants, yet with a number of wild ones too.

Finally, to establish a proposal of local development programme involving WFP, we performed a SWOT analysis [76], with the objective of identifying the strengths, weaknesses, opportunities and threats to the study area. The species relevant for local WFP development (aim iii) have been deduced from the results obtained.

## Results and discussion

### Ethnobotanical description of WFP used

The overall Ripollès ethnobotanical study covers 163 informants along 104 semi-structured interviews covering all 19 municipalities. In the current article, we focused our attention solely on WFP. Information on this subject was provided in 93 interviews involving 143 informants born between 1915 and 1988, 59.44 % women and 40.56 % men.

During the whole research, from 2004 to 2014, which also included cultivated plants, 127 plant taxa were detected as being used for human consumption in the studied region, 80 of which are WFP (62.99 %), and the remaining 47 (37.01 %) -not analysed here- constitute cultivated plants as well as those plant ingredients bought in stores. For information on species information available in one part of the territory under consideration, see [20].

The reported wild food plant species, their vernacular names, information on the frequency of citation, parts used and modes of preparation are shown in Table 1. Taking into account that the vascular flora of the territory investigated consists of approximately 1,600 plant species (J. Vigo pers. comm.) -out of the around 3,700 constituting the flora of the Catalan Countries [70]-, roughly 5 % of them are regarded as WFP in current times.

**Table 1:** Overall results from WFP in the Ripollès district, Pyrenees, by botanical family, including species name, voucher number, vernacular names, part used, mode of consumption and use reports

Botanical family	Taxon	Common names (in Catalan language)	Total UR	Part used	Medicinal use^1^	Mode of consumption	Partial UR
Adoxaceae	*Sambucus nigra* L.^1^ BCN 24984	Bonarbre, sabuc, sabuquer, saüc, saüquer	48	Flower	+	Fried in batter	9
						Water-based beverage	1
				Fruit	+	Boiled in water	1
						Cooked with sugar*	11
						Water-based beverage	9
						Non-fermented sweetened beverage	17
Amaranthaceae	*Chenopodium album* L.^1^ BCN 24751	Blet	1	Aerial part	-	Boiled in water*	1
Amaranthaceae	*Chenopodium ambrosioides* L. BCN 27282	Te	4	Leaf	+	Water-based beverage*	4
Amaranthaceae	*Chenopodium bonus-henricus* L. BCN 27272	Espinac bord, sarrons, sarronets	8	Leaf	-	Boiled in water	3
						Boiled in milk*	1
						Raw	4
Asparagaceae	*Asparagus acutifolius* L.^1,2^ BCN 29976	Espàrrec (young shoot), esparreguera silvestre	2	Young shoot	-	In omelette	2
Amaryllidaceae	*Allium schoenoprasum* L. BCN 28815	Cibulet	1	Bulb	-	In omelette	1
Asparagaceae	*Aphyllanthes monspeliensis* L. BCN 54567	Jonça, junça	5	Flower	-	Raw	5
Apiaceae	*Angelica sylvestris* L.^1^ BCN 24712	Greixa, greixen	15	Leaf	+	Raw	15
Apiaceae	*Apium nodiflorum* (L.) Lag.^1^ subsp. *nodiflorum* BCN 27277	Api bord, àpit	15	Young aerial part	-	Raw*	15
Apiaceae	*Carum carvi* L.^1,2^ BCN 24739	Comí, herba de comí	10	Fruit	+	Condiment*	3
						Fried*	3
						Water-based beverage*	2
						Raw*	2
Apiaceae	*Foeniculum vulgare* Mill.^1,2^ BCN 24888	Fonoll	21	Aerial part	+	Boiled in water	5
						Condiment*	9
						Water-based beverage	1
						In omelette	1
						Raw	3
				Fruit	+	Condiment	2
Apiaceae	*Molopospermum peloponnesiacum* (L.) Koch BCN 24934	Àpit bord, coscoll	42	Young shoot	-	Raw*	42
Apiaceae	*Peucedanum ostruthium* (L.) Koch BCN 24945	Salsufragi	1	Leaf	+	High-grade alcoholic beverage	1
Asteraceae	*Achillea ptarmica* L. subsp. *pyrenaica* (Sibth. ex Godr. in Gren. et Godr.) Rouy BCN 24701	Camamilla	2	Inflorescence	+	High-grade alcoholic beverage*	2
Asteraceae	*Artemisia alba* Turra BCN 24718	Herba de la xocolata, herba menuda	4	Aerial part	+	Condiment	4
Asteraceae	*Cichorium intybus* L.^1,2^ BCN 29660	Endívia, raditxa, xicoia	7	Leaf	-	Raw	7
Asteraceae	*Crepis vesicaria* L. BCN 29719	Raditxa	20	Leaf	-	Raw	20
Asteraceae	*Carlina acanthifolia* All. subsp. *cynara* (Pourr. ex Duby) Arcang. BCN 24738	Carlina, escarlina	16	Inflorescence	-	Preserved in vinegar	1
						Raw*	14
						Boiled in milk	1
Asteraceae	*Cynara cardunculus* L.^×,1,2^ BCN 24759	Herba col, flor d’empresorar (flower)	60	Inflorescence	-	Curd*	60
Asteraceae	*Helianthus tuberosus* L.^×^ BCN 24898	Nyames	7	Leaf	-	Raw	2
				Root	-	Boiled in water*	5
Asteraceae	*Jasonia saxatilis* (Lam.) Guss. BCN 24496	Te de roca	1	Aerial part	+	Water-based beverage	1
Asteraceae	*Leontodon hispidus* L. BCN 24914	Queixalets	3	Young leaf	-	Raw*	3
Asteraceae	*Reichardia picroides* (L.) Roth BCN 113704	Cosconia	1	Leaf	-	Raw	1
Asteraceae	*Sonchus oleraceus* L. BCN 25008	Lletissó	1	Aerial part	+	Raw	1
Asteraceae	*Tanacetum parthenium* (L.) Schultz Bip. BCN 25014	Camamilla borda	1	Inflorescence	+	High-grade alcoholic beverage	1
Asteraceae	*Taraxacum dissectum* (Ledeb.) Ledeb.^1,2^ BCN 25016	Xicoia, xicoina, xicoina de muntanya	87	Leaf	+	Cooked	1
						Raw*	86
Asteraceae	*Taraxacum officinale* Weber in Wiggers BCN 25015	Pixacans	28	Leaf	+	Boiled in water	1
						Raw	26
				Root	-	Condiment	1
Betulaceae	*Corylus avellana* L.^1,2^ BCN 24755	Avellaner	2	Fruit	-	Cooked	2
Boraginaceae	*Borago officinalis* L.^1,2^ BCN 68582	Borratja	3	Leaf	-	Boiled in water*	3
Brassicaceae	*Rorippa nasturtium-aquaticum* (L.) Hayek^1^ BCN 24971	Api bord, berro, creixen, greixen	7	Leaf	-	Raw*	7
Cactaceae	*Opuntia maxima* A.Berger^×^ BCN 46078	Figuera de moro	1	Fruit	-	Raw	1
Caryophyllaceae	*Herniaria glabra* L. BCN 24901	Herba de Noè	1	Aerial part	+	High-grade alcoholic beverage	1
Caryophyllaceae	*Silene vulgaris* (Moench) Garcke BCN 25001	Corretjola, esclafidor	6	Leaf	-	Raw*	6
Convolvulaceae	*Convolvulus arvensis* L. BCN 24753	Corretjola	1	Aerial part	+	Raw*	1
Cupressaceae	*Juniperus communis* L.^1,2^ BCN 24910	Ginebró	2	Fruit	+	High-grade alcoholic beverage	2
Dennstaedtiaceae	*Pteridium aquilinum* (L.) Kuhn BCN 113735	Falguera	1	Frond	+	Raw	1
Ericaceae	*Vaccinium myrtillus* L. BCN 25032	Nabiu (fruit)	16	Fruit		Cooked with sugar*	7
						Raw*	9
Fabaceae	*Trifolium alpinum* L. BCN 25025	Regalíssia	7	Root	+	Raw*	7
Fagaceae	*Castanea sativa* Mill.^×,1,2^ BCN 24740	Castanya (fruit)	2	Fruit	+	Cooked*	2
Gentianaceae	*Gentiana lutea* L.^1^ BCN 24893	Llenciana	5	Root	+	Alcoholic beverage made with wine*	5
Grossulariaceae	*Ribes petraeum* Wulfen in Jacq. BCN 24970	Brosella (fruit), grosellera, ribes	6	Fruit	+	Cooked with sugar	2
						Raw	4
Juglandaceae	*Juglans regia* L.^×,1,2^ BCN 24908	Noguer, nou (fruit), nouer	22	Fruit	+	High-grade alcoholic beverage*	17
						Raw	5
Lamiaceae	*Hyssopus officinalis* L.^1^ BCN 24906	Hisop	1	Flowering aerial part	+	Water-based beverage	1
Lamiaceae	*Lavandula angustifolia* Mill.^1^ BCN 24913	Espígol	1	Aerial part	+	Condiment*	1
Lamiaceae	*Melissa officinalis* L.^×,1^ BCN 24928	Tarongina	6	Aerial part	+	High-grade alcoholic beverage Water-based beverage	4 2
Lamiaceae	*Mentha pulegium* L.^1^ BCN 113598	Poniol	2	Flowering aerial part	+	High-grade alcoholic beverage	2
Lamiaceae	*Mentha spicata* L.^×,1,2^ BCN 24930	Menta	42	Aerial part	+	High-grade alcoholic beverage	1
						Boiled in water	1
						Condiment	3
						Water-based beverage*	5
				Leaf	+	High-grade alcoholic beverage*	2
						Boiled in water*	7
						Condiment*	23
Lamiaceae	*Nepeta cataria* L. BCN 24935	Herba gatera	1	Aerial part	+	High-grade alcoholic beverage*	1
Lamiaceae	*Origanum vulgare* L.^1,2^ BCN 24939	Orenga	60	Flowering aerial part	+	High-grade alcoholic beverage	1
						Condiment*	59
Lamiaceae	*Prunella grandiflora* (L.) Scholler BCN 24956	Herba del traïdor	1	Flower	+	Raw	1
Lamiaceae	*Rosmarinus officinalis* L.^1,2^ BCN 24974	Romaní	12	Aerial part	+	High-grade alcoholic beverage*	2
						Condiment*	1
				Flowering aerial part	+	Condiment	9
Lamiaceae	*Satureja calamintha* (L.) Scheele subsp. *ascendens* (Jord.) Briq.^1^ BCN 24989	Poniol	3	Flowering aerial part	+	High-grade alcoholic beverage*	3
Lamiaceae	*Satureja hortensis* L.^×,1,2^ BCN 29945	Sajolida	14	Flowering aerial part	-	Condiment*	14
Lamiaceae	*Satureja montana* L.^1,2^ BCN 113741	Sajolida	13	Flowering aerial part	+	Condiment	13
Lamiaceae	*Thymus serpyllum* L.^1,2^ BCN 25019	Farigola, farigola borda, farigoleta	5	Aerial part	+	Condiment	3
				Flowering aerial part	+	High-grade alcoholic beverage	1
						Condiment*	1
Lamiaceae	*Thymus vulgaris* L.^1,2^ BCN 25023	Farigola	36	Aerial part	+	Boiled in water	2
						Condiment	13
				Flower	+	Boiled in water	1
				Flowering aerial part	+	Boiled in water*	6
						Condiment*	14
Lauraceae	*Laurus nobilis* L.^1,2^ BCN 24912	Llor, llorer, llaurer	39	Leaf	+	High-grade alcoholic beverage*	2
						Condiment*	37
Liliaceae	*Gagea fistulosa* (Ram. ex DC.) Ker-Gawler BC 639665	Xarvió	1	Bulb	-	Boiled in water	1
Malvaceae	*Malva sylvestris* L.^1^ BCN 24924	Malva	13	Flowering aerial part	+	High-grade alcoholic beverage*	2
				Fruit	-	Raw*	6
				Young shoot	-	Raw	2
				Not reported		Raw	3
Malvaceae	*Tilia platyphyllos* Scop.^×^ BCN 25024	Flor de tell, til·la (both names referring to flowers with bract)	1	Flower with bract	+	High-grade alcoholic beverage	1
Moraceae	*Ficus carica* L.^×,1,2^ BCN 24887	Figuera	3	Infructescense	+	Cooked with sugar	1
						Raw	2
Papaveraceae	*Papaver rhoeas* L. BCN 24940	Rosella, rosella de camp	18	Leaf	-	Raw*	18
Poaceae	*Hordeum distichon* L.^×,1^ BCN 24902	Ordi	2	Seed	+	Boiled in water*	1
						Water-based beverage*	1
Polygonaceae	*Fagopyrum esculentum* Moench^×,1,2^ BCN 24886	Fajol	8	Seed	-	Boiled in water*	8
Polygonaceae	*Polygonum aviculare* L. BCN 24952	Tiravaques	1	Aerial part	-	Raw	1
Polygonaceae	*Rumex acetosa* L.^1^ BCN 27285	Xiscoll	1	Aerial part	-	Raw*	1
Polygonaceae	*Rumex scutatus* L.^1^ BCN 24979	Bedola, madola, verola	16	Leaf	+	Raw*	16
Portulacaceae	*Portulaca oleracea* L.^1^ BCN 24953	Verdolaga	7	Leaf	-	Raw*	7
Rosaceae	*Crataegus monogyna* Jacq. subsp. *monogyna* BCN 24756	Arç blanc	1	Fruit	+	Raw	1
Rosaceae	*Fragaria vesca* L.^2^ BCN 24889	Maduixa (fruit), maduixa de bosc (fruit)	24	Fruit	+	Cooked with sugar Macerated in wine and sugar	1 4
						Raw*	19
Rosaceae	*Prunus spinosa* L. BCN 24958	Aranyoner, arç, arç negre	29	Fruit	+	High-grade alcoholic beverage*	20
						Cooked with sugar*	3
						Raw*	5
				Fruiting aerial part	+	Cooked	1
Rosaceae	*Rosa* sp.^1^	Rosa	2	Flower	+	Raw	2
Rosaceae	*Rosa tomentosa* Sm. BCN 24973	Escanyaguilles, grataculs, roser de bosc, roser bord	6	Fruit	+	Cooked with sugar*	2
						Raw	2
				Not reported		High-grade alcoholic beverage*	2
Rosaceae	*Rubus idaeus* L. BCN 24977	Gerdó (fruit), jordó (fruit), jordonera	28	Fruit	-	High-grade alcoholic beverage	3
						Cooked with sugar*	6
						Raw*	19
Rosaceae	*Rubus ulmifolius* Schott^1^ BCN 24978	Móra (fruit), móra de romeguera (fruit), romeguera	28	Fruit	+	Cooked with sugar*	19
						Raw	9
Rutaceae	*Ruta chalepensis* L.^1^ BCN 24980	Ruda	21	Aerial part	+	Condiment*	21
Scrophulariaceae	*Antirrhinum majus* L. subsp. *majus* BCN 27271	Cadells, gossos	1	Inflorescence	-	Raw	1
Urticaceae	*Urtica dioica* L.^1^ BCN 25030	Ortiga, ortiga major, ortigó	18	Aerial part	+	High-grade alcoholic beverage*	2
						Boiled in water*	1
						In omelette*	1
				Leaf	+	Boiled in water	3
						Condiment	3
						In omelette	8
Urticaceae	*Urtica urens* L.^1^ BCN 25031	Ortiga rènega	1	Leaf	+	In omelette	1
Valerianaceae	*Valerianella locusta* (L.) Laterrade BCN 49861	Canonge, margarita, marieta	8	Leaf	-	Raw	8
			967				967

^1^Reported medicinal use linked to the ingestion of the plant (internal administration only)

^×^Minor crop

^1^Taxon present in [73]. ^2^Taxon present in [74, 75]

*Use published in [20]; this does not mean that the same use has not been detected in the present study (the number of use reports may have increased from the quoted paper)

The four botanical families with more than five species and accounting for 67.94 % of total use reports (UR) included Asteraceae (with 14 species representing 24.61 % of total UR), Lamiaceae (14 spp., 20.37 % of UR), Rosaceae (7 spp., 12.20 % of UR) and Apiaceae (6 spp., 10.75 % of UR). The major families, well known for providing numerous cosmopolitan food plants, contain a high number of Mediterranean representatives. The remaining 30 botanical families accounted for 32.06 % of total UR. The most cited food plant species (those with more than 5 % of total UR) were *Taraxacum dissectum* (9.00 %), *Cynara cardunculus* (6.20 %) and *Origanum vulgare* (6.20 %). These are followed by species with more than 2 % of total UR (in descending order of UR): *Sambucus nigra, Mentha spicata, Molopospermum peloponnesiacum, Laurus nobilis, Thymus vulgaris, Prunus spinosa, Rubus idaeus, Rubus ulmifolius, Taraxacum officinale, Fragaria vesca, Juglans regia, Foeniculum vulgare*, *Ruta chalepensis* and *Crepis vesicaria.*

Different plant parts and modes of preparation of WFP were recorded and grouped according to different categories. The most consumed parts of these plants are the leaves (33.82 % UR), aerial parts (24.41 %), followed by fruits and infructescences—excluding seeds—(22.03 %), and, significantly lower, flowers and inflorescences (10.34 %).

Direct consumption (i.e., raw) accounted for most use reports (42.40 %) mostly in the form of salads, followed by condiments and preservatives (24.20 % UR), high-grade alcoholic beverages (7.55 % UR) mostly in the form of *ratafia* (a local liquor), and curd (6.20 % UR) in order to make cheese. Twelve other minor preparations accounted for the remaining percentage. For greater detail regarding these preparations, see [20].

### Tradition, consistency and reliability of food plant use

Food and culture are very close concepts; for this reason Kittler et al. [77] assert that “eating is a daily reaffirmation of [one’s] cultural identity”.

Out of the 80 WFP recorded in this study, 42 (52.5 %) were already quoted in the 17th century [73] and 22 (27.5 %) are still considered in the present day [74, 75]. The coincidental taxa with those present in these two works are marked in Table 1. Though these data can slightly vary, since the books quoted do not use scientific names and some popular names can be ambiguous, this adds up to a really old tradition in plant use, which is confirmed by it perseverance up to the present day. Even more, if we take into account that in general only the major agricultural and culinary elements are present in the works dealt with. Certainly, above quoted figures represent relatively high percentages of WFP quoted in the present research included in what we could consider the corpus of useful (mostly food) plants in Catalonia at the beginning of the 17th century [73]. This is, undoubtedly, an indication of the long-standing tradition in the folk knowledge regarding plant management since ancient times, this bringing a further element of support to the consistency and reliability of WFP uses in the territory considered. Conversely, the number and percentage of plants here reported included in the compilation of Catalan cooking are, though not insignificant, smaller. This is due, on the one hand, to the fact that we strictly include in our work only wild plants (and a very few minor crops) and in these reference books attention is paid mostly to cultivated plants appearing in cookery recipes. On the other hand, even if the methodology to establish the collection of recipes included field work, only a few particular household informants were interviewed and most information was taken from literature, professional cooks and restaurant menus [74, 75]. Thus, in the first place we can affirm that, even with the above-mentioned constraints, it is relevant that one quarter of the WFP with ethnobotanical information in the territory studied appear in the current corpus of Catalan culinary heritage. Secondly, our results suggest that further updates of this compilation should take more into account the ethnographic approach to collecting information. In this way, its information (of course currently very valuable) would be increased and improved with data on WFP, which are now very biased and restricted to a few spices and allied plants. The authors of the compilation proposed, as future tasks, to acquire a greater knowledge of the condiments and, in general, to assess and identify all culinary products [75]. Clearly, works such as this one presented here may be of value in order to achieve such purposes.

The informant consensus factor (F_IC_, [72]) for WFP in the area considered has a value of 0.92, very close to 1, the maximum possible value. This indicates a high level of consistency of the information reported by the informants, i.e., that their corpus of knowledge on food plants is the reflection of a quite general agreement or an ingrained tradition rather that the expression of incoherent, disperse uses. This value, often calculated in ethnopharmacological works, is not common in research dealing with food plants, but we believe that it is important to assess it in such studies in order to evaluate the reliability of the collected information. In this case, the F_IC_ value is one of the highest in Pyrenean and neighbouring Mediterranean areas studied for medicinal plants ([60] and references therein). It is even higher than the 0.87 found for medicinal plants in one of the three areas now prospected (Higher River Ter Valley; [56]). It reaches a higher value than those calculated for different plant uses in Valais (Alps, Switzerland), ranging from 0.63 to 0.82 [23]. Moreover, it is also higher than those recorded, also for medicinal uses, in some Mexican ethnic groups [78, 79], suggesting that quality ethnobotanical information can also be found in industrialised societies.

Furthermore, 50 out of the 80 WFP quoted in the present work (62.5 %) meet the reliability criterion of having been reported by at least three independent informants [80, 81] (Table 1).

Both F_IC_ and the three-informant minimum were established in order to detect plants that could be good candidates in processes of searching for new sources of plant-based medicines. Similarly, the ancient tradition together with the high reliability and consensus values obtained in this study place the Pyrenean plants mentioned by the informants in good position for further studies aiming to develop food plant (or nutraceutical, see later) products.

### Knowledge on WFP according to gender, age and geographical area of informants

With the ethnobotanical information collected, descriptive analyses were undertaken to explore patterns of WFP knowledge, along with differences amongst participants, according to various demographic variables including gender, age and locality of residence. Results are presented in Table 2.

**Table 2:** Variation on wild food plant and minor crop knowledge according to age, gender and geographical area of informants

Variable	Date of birth			Gender		Geographical area			
	1915–1939	1940–1964	1965–1989	M	F	Higher ter river valley	Higher freser river valley	Lower ripollès	Total
Number of informants	104	35	4	58	85	73	36	34	143
%	*72.73*	*24.48*	*2.80*	*40.56*	*59.44*	*51.05*	*25.17*	*23.78*	*100*
Total species elicited (several may coincide)	71	58	12	62	70	67	46	37	*80*
%	*88.75*	*72.5*	*15*	*77.5*	*87.5*	*83.75*	*57.5*	*46.25*	
Range spp/inf	1 to 17	1 to 17	5 to 7	1 to 17	1 to 17	1 to 17	2 to 17	2 to 12	
Avg. spp/inf	6.06	**8**	6	6.45	6.59	5.66	**8.78**	6.03	6.53
SD spp/inf	3.73	4.43	0.82	4.07	3.87	4.20	3.55	2.74	
Total use reports elicited	647	296	24	388	579	428	332	207	967
%	*66.91*	*30.61*	*2.48*	*40.12*	*59.88*	*44.26*	*34.33*	*21.41*	*100*
Range UR/inf	1 to 17	1 to 17	5 to 7	1 to 17	1 to 17	1 to 17	2 to 17	2 to 12	
Avg. UR/inf	6.22	**8.46**	6	6.69	6.81	5.86	**9.22**	6.09	6.76
SD UR/inf	3.88	4.93	0.82	4.38	4.11	4.45	3.87	2.85	

Values in *italics* indicate percentages. The higher values for some of the variables are in bold

*Abbreviations*: *Avg* average, *SD* standard deviation, *M* male, *F* female, *spp/inf* species/informant, *UR/inf* use report/informant

**Fig. 2:**
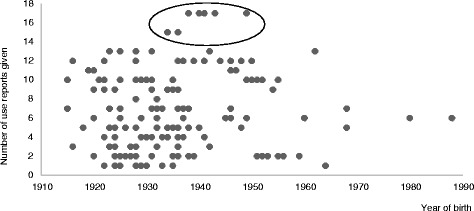
Number of use reports given according to age of informants. Data show a great variability, with a subgroup (born between 1930 and 1950) with relatively higher values (outliers). Average values are given for reference

Among the three age groups assigned, a great variability was found in terms of WFP species elicited, and use reports given showed no clear tendency within the data (Fig. 2). In general, middle aged people (born between 1940 and 1964) elicited both the highest number of species per informant (almost 8), as well as the highest amount of UR per informant (almost 8.78), while younger participants (born between 1965 and 1989) showed an opposite trend, although very few were interviewed within this age group, and cautious conclusions should be drawn for younger informants. Contrarily to what one would expect, older informants, i.e., those born between 1915 and 1939, showed values closer to those of younger informants, corroborating the conclusion amongst various Hotï communities [82], and on Portuguese populations [83] that, in terms of ethnobotanical knowledge, middle aged people can hold the richest, or at least a considerable, pool of biocultural information. One possible explanation for the relatively low performance of the oldest informants is, apart from senility and loss of memory, that they knew famine times (many of them lived, as adults, the Spanish 1936–1939 war and the hard post-war years) in which some wild plants were a compulsory, and not always desired, basic food. One example of this kind of food use was the consumption of the raw immature capitula receptacles of *Carlina acanthifolia* subsp. *cynara* as bread, in post-war periods with scarcity of white bread. So, as we could verify, some of these people did not like to evocate such times and, thus, did not mention these plants. Conversely, slightly younger people (those here named middle-aged) had the knowledge and the experience of these plants, but not with a forcibly negative connotation, since they lived at least in slightly better conditions.

Gender differences were not as sharp as in age groups, there being various explanations that could justify such a fact. On one side, various interviews were undertaken in a same household, where inhabitants share knowledge and practices regarding WFP, yet this would have had the same effect regarding other variables such as age. Another factor may be a rather reduced distinction on gender roles, as farm-related activities havetaken a second place. Nonetheless, such differences indicate a slightly higher report of species and uses by women than by men. This is logical, taking into account that in the studied area (as in most places) women took -and still basically take- care of the houses and of food production in homegardens and preparation [62]. Additionally, the consumption of WFP in the Ripollès district has been linked to agricultural and cattle-raising activities (plants consumed in situ and with minimal or no preparation). In this scenario, children have always been relevant WFP consumers [84], mostly in past times when they accompanied, and sometimes helped, their parents in the field. One very traditional activity was to devote the afternoons of Sunday (the only day in the week at least partly without agricultural work) to the collection of fruits (e.g., of *Fragaria vesca*, *Rubus idaeus*, *R. ulmifolius* or *Vaccinium myrtillus*) for jam, jellies and similar products. This activity is remembered to have been still regularly practiced 40 years ago by one of the authors (TG), native to the studied region. Apart from the fact that this plant use is one of the remnants from this period with a higher persistence nowadays (of course, not forcibly limited to Sunday afternoon), it used to be conducted only by family mothers, so this could be another argument to explain the greater involvement of women in plant food affairs. This agrees with the general situation found in Catalan ethnobotany [9, 11, 20] and with the assertion that the retention and transmission of folk plant management knowledge is mainly linked to women [85–87].

Clear differences amongst geographical areas were detected as much for species elicited as for use reports given per informant (Table 2). Here, inhabitants from the Higher Freser River Valley showed greater knowledge, considerably more than those from either the Higher Ter River Valley or the Lower Ripollès. The most isolated of the three geographical areas constituting the studied district is the Upper Ter River Valley, although results in the latter are closer to the more urbanized and industrialized Lower Ripollès than to the more ethnobotanically rich Higher Freser River Valley. This could be explained by the fact that the Higher Freser River Valley -fairly reliant on local natural resources in previous generations- may have also benefited from ingredients associated with its greater geographical openness and brought by other groups of people.

Hence, in our study, age and geographical area of informants seem to be more relevant to WFP knowledge than gender. Middle-aged people and inhabitants from the Higher Freser River Valley seem to have a greater knowledge on WFP both in relation to the number of species elicited as well as the diversity of uses and preparations reported. To a lesser degree, women seem to have a slightly higher WFP knowledge than men.

### Medicinal uses of WFP

In our preliminary work covering one of the three areas studied here [20], we proposed the term *folk functional food* to refer to those plants (or, when applicable, fungi, animals or other organisms) that have popular uses as food and, at the same time, are claimed to have medicinal properties when ingested (i.e., considering only internal administration). This kind of research confirms the relevance of this concept in ethnobotany [14]. As stated in the introduction, there is a large agreement in the relevance of nutraceutical products from an ethnobiological provenance. In the present work, 73.75 % (59 out of 80) of the WFP reported locally have medicinal applications used internally (Table 1). This result is much higher than the 33.8 % reported in the preliminary work [20], confirming not only the Hippocratic classical assertion that “your food shall be your medicine” [88], but also the richness in plants of the studied region, for furthering cutting-edge research linked to these products–placed in the interface between nutrition and health.

### Potential species for a future local WFP development programme

With a nuanced study of demographic and agroculinary factors, better biocultural revitalization programmes may be achieved in rural areas. A preliminary strengths, weaknesses, opportunities and threats analysis (SWOT, [89, 90]) is provided (Table 3), in order to depict the situation of the topic addressed and establish the mechanisms of facilitating a WFP promotion programme.

**Table 3:** Strengths, weaknesses, opportunities and threats (SWOT) analysis for the establishment of a local development programme involving WFP

Element	Positive	Negative
Internal (to the study area)	Strengths	Weaknesses
	- Knowledge and use of WEP still present in the region. - Relatively low costs of production - Complements diet. - Revalorization of WEP use in local restaurants, rural tourism and others. - Cooperation amongst residents. - Conservation and development of biocultural diversity. - Proximity products’ local fairs.	- Demographic decrease towards urban areas. - Geographical dispersion. - Abandonment of farms. - Lack of permanent jobs. - Overexploitation of certain species (e.g., *Molopospermum peloponnesiacum*).
External	Opportunities	Threats
	- Revalorization of WEP use on a regional scale. - Fairs at the regional/national level. - Regulatory systems and certifications such as designations of origin, geographical indications and traditional specialities guaranteed. - Public and private programmes for recovering old varieties and WEP use - Replanting of overexploited species along with study of germination and cultivation for conservation purposes.	- Short optimal season, with harsh weather during winter. - Random cases of poisoning. - Changes in gastronomic trends.

In general, species and modes of preparation with higher reports tend to be those most preferred in an area, hence having a higher potential for successful local development. In the Ripollès district (Table 4), the three species with UR higher than 5 %, i.e., *Taraxacum dissectum*, *Cynara cardunculus* and *Origanum vulgare* are herbs, with leaves (and aerial parts) being the most important part used, followed by inflorescences. Most preferred modes of preparation for such relevant species included “none” (eaten raw), followed by “as curd” and “condiment”. Most probably, adapting such recipes to current settings would enable revitalization.

**Table 4:** Most-preferred species (%UR > 5), plant parts used, and modes of preparation

Species (Family)	Use reports (UR) (%)	Plant part used (%)	Mode of preparation (%)	
*Taraxacum dissectum* (Asteraceae)	87 (9.00 %)	**Leaf (100 %)**	***Raw (98.85 %)***	*Cooked (1.15 %)*
*Cynara cardunculus* (Asteraceae)	60 (6.20 %)	**Inflorescence (100 %)**		***Curd (100 %)***
*Origanum vulgare* (Lamiaceae)	60 (6.20 %)	**Flowering aerial part (100 %)**	***Condiment (98.33 %)***	*HG alcoholic bev. (1.67 %)*

Plant parts used and modes of preparation in bold represent those values with highest significance for each species

*Abbreviations*: *HG alcoholic bev.,* high-grade alcoholic beverage

To facilitate the success of any perspective in WFP development programme, targeting to informants with higher potential for promoting production, exchange and consumption should be favoured. In addition, taking into consideration differential preferences for distinct WFP could facilitate a stratification of local WFP promotion which would further ensure acceptability in different target groups. Moreover, the large number of WFP mentioned in the course of this study could provide interesting opportunities for further diversification of mountain agriculture and the cultivation of alpine plants, representing a new market niche for this kind of agriculture [2, 23]. The possibility of finding this kind of WFP in vegetable markets, and a lot of other initiatives to strengthen these wild vegetables, could be a good opportunity to combine aspects of tradition with elements of innovation [29]. Given the relevancy of the use of *Cynara cardunculus* as milk curd in the area (60 UR, Table 1), an initiative of cultivation and use to elaborate traditional cheese on a small or medium industrial scale could be envisaged, similar to the one undertaken in the nearby district of Alt Empordà [11], confirming this taxon to be what Duke and DuCellier [91] termed as an alternative cash crop.

Another element involving the knowledge on WFP in economic issues is the potentiation of some incipient current uses, such as the presence of some of the WFP in the menus of various restaurants. This is already the case for *Fagopyrum esculentum* and *Taraxacum dissectum*, and could be enlarged to other taxa, in the frame of the increasing interest in proximity cuisine, rural tourism and related aspects. As has been shown in other industrialised countries, WFP may play a relevant role in the socioeconomic regeneration of rural societies [6]. People in the Pyrenean region involved in the present study eat one plant, *Rumex scutatus* (quoted with a high number of UR, 16, Table 1), for its particular acid flavour. This species belongs to the same genus and has very similar properties to *R. acetosa* (also reported, though only once, in the studied area, Table 1), a plant mentioned in the introduction to have been the origin, on an ethnobotanical basis, of a fundamental dish of *nouvelle cuisine*. This suggests a general similar background in the patterns of plant knowledge, consideration, management and use by human beings in different areas.

It is without doubt that all uses issuing from a possible WFP promotion project should be sustainable. On this point, data furnished by our informants is relevant too. Concerning one of the favourite salad plants in the area (*Molopospermum peloponnesiacum*, with 42 use reports; Table 1), some interviewees entertain the idea that the collection of this plant, locally and temporarily really important, may help its conservation. Should the use of this species be promoted, an assessment of conservation questions would need to be carried out, and possibilities of cultivation should be considered.

## Conclusion

According to our study, about a 5 % of the local flora corresponds to WFP, indicating the relatively large amount of WFP used in the Ripollès district and their associated ethnoculinary knowledge. Nonetheless, consumption is perceived to be in recession, with the abandonment of certain species, preparations and recipes, or these being substituted by newer trends. Catalonia is, together with Japan, one of the few countries (in fact these are the only ones, according to Institut Català de la Cuina [75]) to have built, on an ethnographic basis, a comprehensive inventory of traditional culinary recipes and, as has been seen, the presence of plants (even WFP) in the kitchen is not at all residual. In any case, and conversely to what this decline in use could make us think, some indices show a strong degree of tradition, consistence and reliability of the food plant knowledge in the regions studied.

The large number of WFP in the area considered and in other European mountain territories (see works quoted in the introduction) testifies to the importance of the use of wild food greens in this continent, at a similar rate to those in other geographical areas, where they have been classically considered very relevant, as is the case of the so-called *quelites* in Mexico [92]. This offers an arsenal of products open to further research in order to better feed the World’s population.

At a time when natural and cultural resources are being re-valued and promoted, further studies analysing *demographic* factors and ethnobotanical change may shed light on the strategies, strengths and shortcomings of the promotion of biocultural diversity. Compensations could be as diverse as the consumption, in itself, of interesting products, a source for small, complementary incomes, recreation, or just enjoying being close to nature [93]. In addition, a combination of tradition aspects with innovation elements may constitute a key for the survival and reformulation of the relations between humans and plants in the Catalan Pyrenees. Studies such as the one presented herein may be significant to establish such knowledge and practices, along with the characterization of those demographic and agroculinary factors that contribute to the promotion of local WFP production, consumption and exchange, in the frame of a revitalisation of mountains, meadows, and in general, rural areas.

## Abbreviations

Avg: Average
BCN: Herbarium of the *Centre de Documentació de Biodiversitat Vegetal, Universitat de Barcelona*
F: Female
F_IC_: Informant consensus factor
HG alcoholic bev.: High-grade alcoholic beverage
M: Male
spp/inf: species/informant
SWOT: Strengths, weaknesses, opportunities and threats
SD: Standard deviation
UR: Use reports
UR/inf: Use report/informant
WFP: Wild food plants (including semi-wild plants and minor crops)
